# Maternal bovine appeasing substance administration at castration reduces stress, pain-related behavior, and respiratory disease in feedlot bulls

**DOI:** 10.1093/tas/txag062

**Published:** 2026-05-09

**Authors:** Martin Zinicola, Reinaldo F Cooke, Juan Manuel Bollatti, Ramiro Andreola

**Affiliations:** Cowix, San Francisco, Córdoba, Argentina; Department of Animal Science, Texas A&M University, College Station, TX, 77843, United States; Centro Experimental de Nutrición Animal Biofarma, Córdoba, Córdoba, Argentina; Centro Experimental de Nutrición Animal Biofarma, Córdoba, Córdoba, Argentina

**Keywords:** bovine appeasing substance, castration, feedlot bulls, stress

## Abstract

This experiment evaluated behavioral, physiological, and health responses of feedlot-adapted growing bulls administered the maternal bovine appeasing substance (**mBAS;** FerAppease, FERA Diagnostics and Biologicals; College Station, TX) at the time of castration. Angus-influenced (*n* = 182) bulls were purchased from 3 sources with no previous health and management history, and transported to the research center on d −40. Bulls were assigned to initial processing and revaccination on d −36 and −12, respectively. Bulls were ranked by body weight (**BW**) on d −12 (284 ± 3 kg) and source, and assigned to 1 of 26 pens (*n* = 7 bulls/pen, 13 pens/treatment) on d 0. Pens were randomly assigned to receive bulls administered 10-mL of mBAS or no treatment (**CON**) immediately prior to band-castration (d 0). The mBAS was applied topically on the nuchal skin area (5 mL) and above the muzzle (5 mL) and reapplied on d 14. Final BW was recorded on d 42, whereas feed intake and signs of bovine respiratory disease (**BRD**) were recorded daily. Hair samples from the tail-switch were collected on d 0 and 14. Subjective pain-related behaviors were evaluated daily from d 0 to 13 using 3-point scoring system (0 = no pain, 1 = moderate pain, 2 = severe pain). Bulls were also fitted with an ear tag (CowManager, Agis Automatisering BV, Harmelen, The Netherlands) to record behavioral responses. No treatment effects were detected (*P* ≥ 0.41) for BW gain, feed intake, and gain: feed, whereas cattle administered mBAS tended to have greater (*P* = 0.08) final BW. Hair cortisol concentrations did not differ (*P* = 0.66) between treatments on d 0, but were less (*P* = 0.02) in mBAS cattle on d 14 (treatment × day; *P* = 0.04). Incidence of BRD signs and mean pain score were less (*P* ≤ 0.05) for mBAS cattle. Cattle administered mBAS spent less time (*P* ≤ 0.03) being not active on wks 1 to 4, spent more time (*P* ≤ 0.03) being highly active during wks 1 and 2, and spent more time (*P* = 0.03) ruminating on wk 3 (treatment × day; *P* ≤ 0.04). Liveweight gain from d 0 to 42 tended (*P* = 0.09) to be greater for mBAS pens, and final liveweight was greater (*P* = 0.05) in mBAS pens. Pens that received mBAS had greater (*P* = 0.05) final value and tended (*P* = 0.08) to have greater profit. Administering mBAS to feedlot-adapted bulls at the time of castration attenuated chronic stress, reduced behavioral expression of pain, decreased the incidence of BRD signs, and improved pen-based productivity during a 42-d post-procedural period.

## Teaser

Castration of feedlot-adapted bulls is a relevant welfare and health challenge in beef systems where bulls are castrated after feedlot entry. This study evaluated maternal bovine appeasing substance administration at castration as a strategy to mitigate stress and improve post-procedural health outcomes under commercial feedlot conditions.

## Introduction

Castration is a common management practice in beef production systems to reduce aggressive and sexual behavior, facilitate handling, and improve carcass quality and beef palatability (Bretschneider 2005; Coetzee 2013). However, castration induces inflammatory and neuroendocrine stress responses characterized by pain-related behavioral changes, which result in impaired growth performance and increased susceptibility to bovine respiratory disease (**BRD**; Fisher et al. 2001; Ting et al. 2003; Stafford and Mellor 2011). These negative responses are exacerbated as age and body weight (**BW**) at castration increase, resulting in greater performance losses and prolonged recovery compared with calves castrated early in life (Bretschneider 2005).

In many South American beef production systems such as Argentina, male calves are often backgrounded as intact bulls to maximize growth efficiency and then castrated after feedlot entry to meet market requirements for steers at slaughter (Zone and Galotta 2013). Hence, castration is frequently performed in heavier cattle in these systems, and the stress from castration causes greater disruption to feed intake and growth performance in older animals compared with younger cohorts (Fisher et al. 2001; Bretschneider 2005; Stafford and Mellor 2011). Strategies that mitigate stress and accelerate recovery following castration are thus warranted for feedlot-adapted bulls.

The maternal bovine appeasing substance (**mBAS**) is a synthetic analogue of the natural pheromone secreted by cows, which promotes cow-calf bonding through olfactory recognition by the offspring (Cappellozza and Cooke 2022). Administration of mBAS improved performance and health responses in beef cattle during stressful management events such as weaning and feedlot receiving (Pickett et al. 2024; Cooke et al. 2025; Gellatly et al. 2025). Cappellozza and Cooke (2022) reported that mBAS administration at the time of castration improved post-procedural growth performance in *Bos indicus* bulls; however, the behavioral, physiological, and health-related mechanisms that may explain these responses were not evaluated. Based on this rationale, we hypothesized that bulls receiving mBAS would have improved welfare indicators and health outcomes following castration. To test this hypothesis, this experiment evaluated the effects of mBAS administration at castration on behavioral, physiological, and health responses of feedlot-adapted bulls during the 42-d post-procedural period under commercial conditions.

## Materials and methods

This experiment was conducted at the Centro Experimental de Nutrición Animal Biofarma (Córdoba, Argentina) from August to October 2025. All animals were cared for in accordance with acceptable practices and experimental protocols reviewed and approved by the Ethics and Safety Advisory Committee of the National University of the Litoral, Faculty of Veterinary Sciences (approval no. FCV-1260279-25)

### Animals and treatments

Angus-influenced (*n* = 182) non-castrated bulls were purchased from 3 different sources (2 commercial auction facilities and 1 backgrounding ranch) and used in this experiment. No information regarding previous health and management history was available. Bulls arrived at the experimental facility on d −40 and were allowed a 4-d resting period before initial processing on d −36, which consisted of BW assessment (239 ± 3 kg), vaccination against respiratory pathogens (Providean Respi 8 Querato; Tecnovax, Buenos Aires, Argentina) and clostridial diseases (Providean Clostridial 10P; Tecnovax), as well as anthelmintic (Bayverm PI; Elanco Animal Health, Indianapolis, IN, USA) and ectoparasite control (Vermectín L.A. Premium; Over, Santa Fe, Argentina). On d −12, bulls were weighed (284 ± 3 kg), revaccinated against respiratory pathogens (Providean Respi 8 Querato; Tecnovax) and clostridial diseases (Providean Clostridial 10P; Tecnovax), and fitted with an ear tag (CowManager, Agis Automatisering BV, Harmelen, The Netherlands) to record behavioral responses including time spent ruminating, eating, and being physically active or inactive (Harvey et al. 2024).

On d 0 of the experiment, bulls were ranked by BW recorded on d −12, source, and assigned to 1 of 26 pens containing 7 bulls each, in a manner that all pens had equivalent BW and the same proportion of bulls from the 3 sources. Pens were randomly assigned to receive bulls administered mBAS (FerAppease; FERA Diagnostics and Biologicals; College Station, TX; *n* = 13 pens, 91 bulls) or no treatment (**CON**, *n* = 13 pens, 91 bulls) at the time of band-castration on d 0 (Callicrate Pro-Bander^TM^; No-Bull Enterprises, LLC St. Francis, KS). More specifically, bulls were processed for BW assessment and segregated by treatment (2 groups), and immediately processed again and restrained in the squeeze chute for castration and treatment administration. Bulls assigned to CON were processed and castrated first to avoid cross-contamination during treatment application (Colombo et al. 2020). After castration and treatment administration on d 0, cattle were allocated to their respective pens for a 42-d experimental period. The mBAS was reapplied on d 14 to maintain its biological activity throughout the post-castration recovery period, based on its estimated duration of action (∼15 d; Pickett et al. 2024) and the prolonged nature of stress responses following castration (Fisher et al. 2001; Ting et al. 2003; Stafford and Mellor 2011). The active ingredient of mBAS is based on a mixture of fatty acids including palmitic, oleic, and linoleic acids, added at 10% of the excipient and estimated to remain in treated animals for 15 d (Pickett et al. 2024). The mBAS (10 mL) was applied topically to the nuchal skin area (5 mL) and above the muzzle (5 mL) using the applicator provided by the manufacturer (FERA Diagnostics and Biologicals), and according to manufacturer recommendations and previous research (Pickett et al. 2024; Cooke et al. 2025; Gellatly et al. 2025).

Pens were located in an enclosed barn with slatted flooring (6 × 5 m). Pens were arranged in four rows containing 6 or 7 pens/row, and rows were alternately assigned to mBAS and CON pens to preserve distance between pens from different treatments (Colombo et al. 2020). Rows were evenly distributed across the facility to reduce potential confounding effects related to pen location, handling sequence, airflow, and other environmental sources of variation. From d −40 to 42, cattle had ad libitum access to water and were offered a total mixed ration (**TMR**; Table 1) once daily at 1000 h. Feed delivery was adjusted to achieve approximately 10% orts (as-fed basis). The 42-d experimental period was selected to encompass the acute and subacute post-castration period, during which stress, behavioral, health, and early productivity responses are expected to be most evident (Bretschneider 2005; Stafford and Mellor 2011; Tschoner and Feist 2022).

**Table 1:** Composition and nutritional profile of the total mixed ration offered for ad libitum consumption to feedlot cattle during the experiment.^a^

Item	A	B	C	D
***Composition, dry matter basis***				
*** Cracked corn, %***	21	20	22	35
*** Steam-flaked corn, %***	13	21	26	0
*** Corn silage, %***	0	0	15	20
*** Wet distillers’ grains, %***	25	21	22	16
*** Alfalfa hay, %***	30	20	4	16
*** Peanut hulls, %***	8	15	8	10
*** Supplement mix^b^, %***	3	3	3	3
***Nutritional profile,^c^ dry matter basis***				
*** Net energy for maintenance, Mcal/kg***	1.77	1.81	1.97	1.72
*** Net energy for gain, Mcal/kg***	1.13	1.16	1.31	1.11
*** Total digestible nutrients, %***	73.7	74.2	80.0	71.3
*** Neutral detergent fiber, %***	36.0	35.0	30.0	34.1
*** Crude protein, %***	16.5	14.5	14.9	15.0

a Diet A was offered from d –40 to –37, diet B was offered from d –36 to –31, diet C was offered from d –30 to –1, and diet D offered from d 0 to 42.

b Diet A and B: containing 15% Ca, 20% NaCl, 370 mg/kg Cu, 8 mg/kg Se, 1625 mg/kg Zn, 85,000 IU/kg of vitamin A, 16,630 IU/kg of vitamin D3, and 120 IU/kg of vitamin E, 1260 mg/kg of monensin (37.8 g/ton of total mixed ration, dry matter basis). Diet C and D: containing 20% urea, 18% Ca, 21% NaCl, 370 mg/kg Cu, 8 mg/kg Se, 1625 mg/kg Zn, 91,104 IU/kg of vitamin A, 18,221 IU/kg of vitamin D3, and 123 IU/kg of vitamin E, 1260 mg/kg of monensin (37.8 g/ton of total mixed ration, dry matter basis), and 160 mg of diflubenzuron.

c Based on wet chemistry procedures by a commercial laboratory (Feedlab Biofarma; Córdoba, Argentina). Net energy for maintenance and gain were calculated using the equations for energy value of feeds described by NASEM (2016).

### Sampling

Samples of the TMR were collected weekly, pooled across weeks, and analyzed for nutrient content (Feedlab Biofarma; Córdoba, Argentina). Feed intake [dry matter (**DM**) basis] was evaluated from d 0 to 42 by recording offered and non-consumed TMR daily. Samples of the offered and non-consumed TMR were dried at 105°C for 24 h to determine DM content. Feed intake of each pen was divided by the number of cattle within each pen, and expressed as kg per animal/day on DM basis. Full BW was recorded on d 0 prior to castration and on d 42 for average daily gain (**ADG**) calculation. Moreover, cattle were weighed prior to the first feeding of the day to minimize variation associated with gut fill (Colombo et al. 2019). Gain to feed (**G : F**) ratio was calculated using ADG and feed intake from each pen.

Hair samples were collected from the middle portion of tail switch from all cattle on d 0 prior to castration and on d 14 prior to treatment re-application. For samples collected on d 0, a section of hair approximately 20 cm long extending distally from the midpoint of the tail was clipped using electric clippers. From this clipped area, the first 1-cm segment of hair closest to the skin (proximal segment) was collected for analysis. For samples collected on d 14, hair was sampled from the exact same location where samples from d 0 were collected. During this second sampling, only the hair that had grown between d 0 and d 14 was collected, allowing the analysis of hair growth corresponding to cortisol accumulation during this 14-d period. After collection, hair samples were stored at −20°C until analysis. The 14-d interval between samplings for analysis of hair cortisol concentrations is based on the known growth rate of tail switch hair (∼1 cm per 15 d; Burnett et al. 2014), allowing assessment of cortisol accumulation over the 14-d interval (Schubach et al. 2017; 2020; Pickett et al. 2024). Hair samples were not collected later in the experiment because cattle were processed again on d 42, which would result in a 28-d interval between samplings.

### Behavioral, health, and pen-based productivity assessments

Subjective pain-related behaviors were evaluated daily from d 0 to 13 using a scoring system adapted from Gleerup et al. (2015). Briefly, observations were conducted by a single trained evaluator blinded to treatment assignments. A 3-point scale (0 = no pain, 1 = moderate pain, or 2 = severe pain) was used to assess five behavioral indicators in each animal: attention, head position, ear and back position, response to human approach, and facial expression. Scores of 0 represented normal behavior or posture, whereas scores of 2 represented pronounced deviations consistent with pain-related behavior, including reduced attention to the surroundings, lowered head position, drooped ears, tense facial expression, reduced response to human approach, and arched back posture (Gleerup et al. 2015). Scores of 1 represented mild or intermittent deviations from normal behavior or posture. The scores of each behavioral indicators were averaged daily to generate continuous pain score for each animal. Results from the CowManager system were recorded from d −7 to 42, and averaged by week to facilitate analysis (Harvey et al. 2024). Data recorded during sampling days were discarded to eliminate the confounding effects of gathering, handling, and processing on physical activity.

Cattle were observed daily for BRD signs including depression (lethargy, drooping ears), abnormal posture (gauntness, orthopnea), and altered respiratory character (nasal discharge, dyspnea). Animals diagnosed with these BRD signs received meloxicam (0.5 mg/kg of BW; Metacam, Boehringer Ingelheim, Ingelheim, Germany) and were assessed for rectal temperature. Cattle classified as febrile (rectal temperature ≥ 39.5°C) also received tulathromycin (2.5 mg/kg BW; Draxxin, Zoetis, Buenos Aires, Argentina) as their first antimicrobial therapy. Treatment failure was defined as the need for retreatment based on BRD signs within 14 d of the initial therapy. Florfenicol (40 mg/kg BW, SC; Nuflor, Merck Animal Health, Madison, NJ) was administered as second antimicrobial therapy. Cattle requiring a third antimicrobial therapy received enrofloxacin (7.5 mg/kg BW, SC; Baytril, Bayer Animal Health, Shawnee Mission, KS).

Total liveweight/pen was calculated by summing the BW of cattle within each pen on d 0 and at the end of the experiment (d 42). Hence, final BW of animals excluded from the experiment (mortality) was considered zero (Pickett et al. 2024). A pen-basis economical evaluation was performed according to recent U.S. cattle values (USDA-AMS 2026) including liveweight of each pen ($9.30/kg for initial and $8.81/kg for final liveweight value, respectively), and total feed used by each pen ($300/metric ton). Cattle excluded from the experiment did not contribute to final pen value (final BW = 0), thus incorporating the economic impact of mortality into the analysis. Medication costs used for first antimicrobial therapy were $22 when fever was diagnosed and $12 when fever was not diagnosed. Costs for second and third antimicrobial therapies were $35 and $9, respectively. The labor required for mBAS application was considered negligible, as treatments were administered during routine handling procedures and did not require additional processing time. Profit of each pen was estimated as final liveweight value – (initial liveweight value + total feed costs + total medication costs).

### Laboratory analyses

All TMR samples were analyzed by wet chemistry procedures for concentrations of crude protein (method 984.13; AOAC 2006), acid detergent fiber (method 973.18 modified for use in an Ankom 200 fiber analyzer, Ankom Technology Corp., Fairport, NY; AOAC 2006), and neutral detergent fiber using a-amylase and sodium sulfite (Van Soest et al. 1991; modified for use in an Ankom 200 fiber analyzer, Ankom Technology Corp.). Nutritional profile of TMR is described in Table 1. Net energy for maintenance and gain of the TMR were calculated using the equations for energy value of feeds described by NASEM (2016).

Cortisol was extracted from individual hair samples as previously described (Moya et al. 2013; Schubach et al. 2017). Briefly hair samples were cleaned with warm water (37°C) for 30 min, and dried at room temperature for 24 h. Hair samples were then washed twice with isopropanol, dried at room temperature for 120 h, and ground in a 10-mL stainless steel milling cup with a 12-mm stainless steel ball (Retsch Mixer Mill MM400 ball mill; Retsch, Hannover, Germany) for 5 min at a frequency of 30 repetitions/s. Twenty mg of ground hair and 1 mL of methanol were combined into a 7-mL glass scintillation vial, sonicated for 30 min, and incubated for 18 h at 50°C and 100 rpm for steroid extraction. Upon incubation, 0.8 mL of methanol was transferred to a 2-mL microcentrifuge tube and evaporated at 45°C. Samples were reconstituted in 100 μL of phosphate-buffered saline and stored at −80°C, until analyzed for cortisol concentrations using an enzyme-linked immunoassay cortisol kit (Salimetrics Expanded Range, High Sensitivity 1-E3002, State College, PA). The intra- and inter-assay CV were 2.17 and 2.77%, respectively.

### Statistical analysis

Data were analyzed as a completely randomized design, using pen as the experimental unit and Satterthwaite approximation to determine the denominator degrees of freedom for tests of fixed effects. The MIXED procedure of SAS (SAS Inst. Inc., Cary, NC) was used to analyze quantitative data including growth performance (BW, ADG, feed intake, G : F), hair cortisol concentration, behavioral responses (pain score and CowManager data), and pen-based productive responses. All quantitative data were analyzed for normality using the Shapiro-Wilk test, whereas pain score required log-transformation to achieve normality (W > 0.90). Pain score data were analyzed as continuous variables after log_10_ transformation to meet model assumptions. The GLIMMIX procedure of SAS (SAS Inst. Inc.) with a binomial distribution and logit link function was used to analyze health responses (BRD incidence, mortality). Pen-based assessments used pen(treatment) as random variable, whereas all other models included pen(treatment) and animal(pen) as random variables. Model statements for BW parameters, feed efficiency, and morbidity-related results contained the effects of treatment. Model statements for feed intake, cumulative BRD incidence, and behavioral assessments contained the effects of treatment, time, and the resultant interaction. The specified term for all repeated statements was time, with pen(treatment) as subject for pen-based assessments, and animal(pen) as subject for all other analyses. The covariance structure used was first-order autoregressive, which provided the smallest Akaike information criterion and hence the best fit for all variables analyzed. All results are reported as least square means and were separated using least square difference. Significance was set at *P* ≤ 0.05 and tendencies were determined if *P* > 0.05 and ≤ 0.10. Repeated measures are reported according to main treatment effect if the treatment × time interaction was *P* > 0.10.

## Results and discussion

### Performance responses

**Figure 1:**
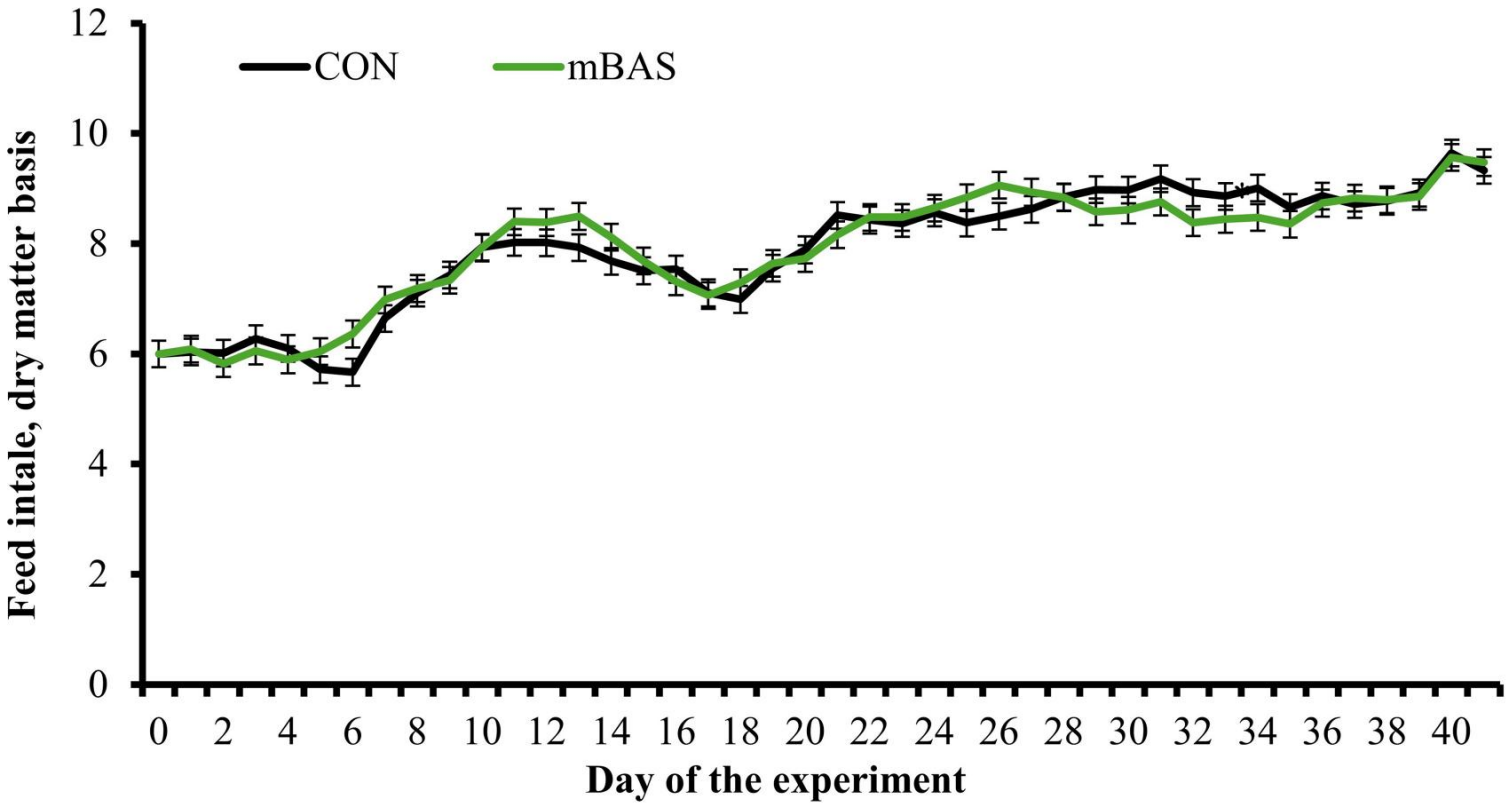
Feed intake during a 42-d feedlot period of bulls administered the maternal bovine appeasing substance (**mBAS**; *n* = 13; FerAppease; FERA Diagnostics and Biologicals; College Station, TX) or not (**CON**; *n* = 13) at the time of band-castration (d 0). the mBAS was applied topically to the nuchal skin area (5 mL) and above the muzzle (5 mL) of each bull, and reapplied on d 14 of the experiment. Feed intake was evaluated by recording feed offer and refusals daily from each pen (dry matter basis), which was divided by the number of animals within each pen and expressed as kg per animal/d. No treatment × day interaction nor treatment effect were detected (*P* ≥ 0.40).

Initial BW did not differ between CON and mBAS cattle (*P* = 0.22; Table 2). Cattle administered mBAS tended to have greater (*P* = 0.08) final BW compared with CON (Table 2). However, ADG did not differ (*P* = 0.41) between treatments (Table 2). No treatment effect (Table 2) or treatment × day interaction (Fig. 1) was detected (*P* ≥ 0.40) for feed intake. Gain: feed during the 42-d experimental period did not differ (*P* = 0.57) between treatments (Table 2). Previous research reported growth performance benefits when mBAS was administered to cattle exposed to stressful procedures such as weaning (Cooke et al. 2020; Schubach et al. 2020; Gellatly et al. 2025), feedlot arrival, and initial processing (Colombo et al. 2020; Cooke et al. 2025). Cappellozza and Cooke (2022) described that *Bos indicus* bulls (∼274 kg of BW) receiving mBAS at the time of castration had greater BW 30 d later compared with bulls not administered mBAS. These studies are consistent with the tendency for greater final BW observed herein, although this outcome should be interpreted with caution because the increased final BW in mBAS cattle was observed without treatment differences in initial BW, ADG, feed intake, and G : F. Nonetheless, numerical differences in initial BW and ADG likely contributed to the tendency for treatment effects on final BW, whereas no treatment differences are observed when initial BW was included as covariate in the final BW analysis (327.2 vs. 328.9 kg for CON and mBAS cattle; SEM = 1.0). The focus of this experiment, however, was to evaluate behavioral, physiological, and health-related outcomes from mBAS administration at the time of castration. The individual growth performance results will not be further discussed in this manuscript.

**Table 2:** Performance parameters during a 42-d feedlot growing period of beef cattle administered the maternal bovine appeasing substance (**mBAS**; *n* = 13) or not (**CON**; *n* = 13).^a^

Item	CON	mBAS	SEM	*P-*Value
***Body weight,^b^ kg***				
*** d 0 (initial)***	306.6	308.7	1.2	0.22
*** d 42 (final)***	326.4	329.7	1.2	0.08
*** Average daily gain, kg/d***	0.471	0.500	0.023	0.41
***Feed intake,^c^ kg/d (dry matter basis)***	7.88	7.90	0.11	0.89
***Gain to feed,^d^ kg/kg***	0.062	0.065	0.003	0.57

a Non-castrated bulls were purchased from 3 different sources with no previous health and management history, and transported to the research center on d –40. Bulls were assigned to initial processing and revaccination on d –36 and –12, respectively. Prior to initial castration on d 0, bulls individually received 10-mL of the mBAS (FerAppease; FERA Diagnostics and Biologicals; College Station, TX) or no treatment (CON). Treatments were applied topically to the nuchal skin area (5 mL) and above the muzzle (5 mL). Bulls were castrated using band-castration (Callicrate Pro-Bander^TM^; No-Bull Enterprises, LLC St. Francis, KS). The mBAS was reapplied to respective steers on d 14 of the experiment.

b Body weight (**BW**) recorded are unshrunk.

c Feed intake was evaluated by recording feed offer and refusals daily from each pen, which was divided by the number of animals within each pen and expressed as kg per animal/d.

d Calculated using average daily gain and feed intake of each pen.

### Hair cortisol and health responses

**Figure 2:**
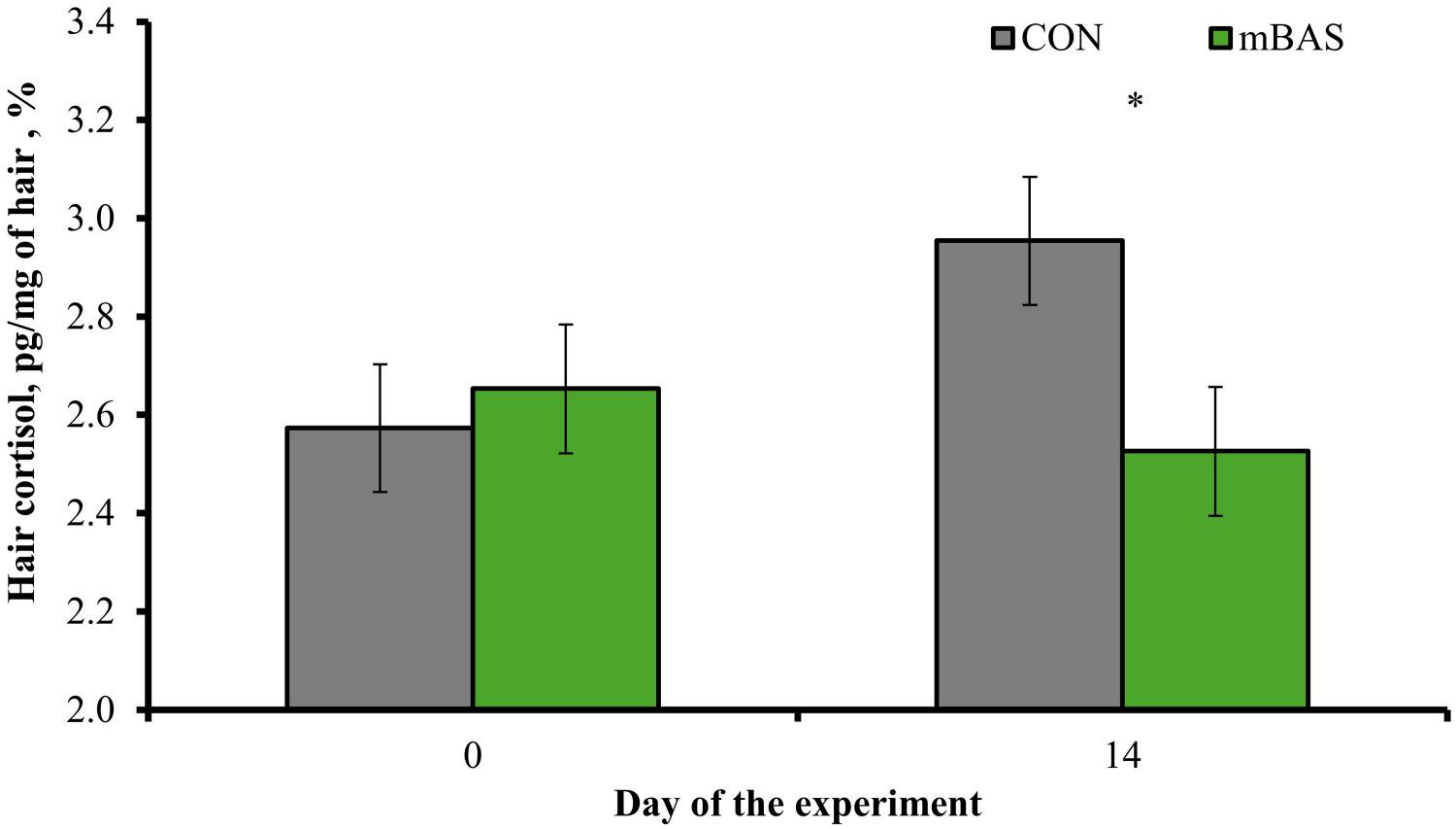
Hair cortisol concentration in cattle administered the maternal bovine appeasing substance (**mBAS**; *n* = 13; FerAppease; FERA Diagnostics and Biologicals; College Station, TX) or not (**CON**; *n* = 13) at the time of band-castration (d 0). the mBAS was applied topically to the nuchal skin area (5 mL) and above the muzzle (5 mL) of each bull, and reapplied on d 14 of the experiment. Hair samples were collected on 0 and 14 prior to treatment administration as in Schubach et al. (2017). a treatment × day interaction was detected *(P* = 0.04). Within days, * *P* = 0.02.

A treatment × day interaction was detected *(P* = 0.04) for hair cortisol concentrations, which did not differ (*P* = 0.66) between treatments on d 0 but were less (*P* = 0.02) in mBAS compared with CON cattle on d 14 of the experiment (Fig. 2). Hair cortisol concentration from the tail switch is a biomarker of chronic stress (Burnett et al. 2014; Moya et al. 2015), as cortisol is progressively incorporated into the growing hair shaft and reflects long-term adrenocortical activity (Moya et al. 2013). Therefore, mBAS administration lessened chronic stress caused by castration based on treatment differences for hair cortisol on d 14 of the experiment. In fact, hair cortisol concentration increased (*P* = 0.03) from d 0 to 14 in CON cattle, but did not change (*P* = 0.43) for mBAS cattle, suggesting that mBAS administration prevented the chronic stress response elicited by the pain of castration (Stafford and Mellor 2011). Pickett et al. (2024) also reported less hair cortisol concentration on d 14 and 28 following band-castration in high-risk steers (i.e., steers at a higher risk of BRD due to recent weaning, commingling, transport stress, and unknown health history; Duff and Galyean 2007) administered mBAS compared with placebo-treated cohorts. These authors also described that mBAS steers had less mean plasma cortisol concentrations during the initial 4 h following band-castration compared with placebo cohorts. Collectively, results from this experiment and Pickett et al. (2024) support mBAS administration as a strategy to attenuate the acute and chronic stress responses associated with castration.

**Figure 3:**
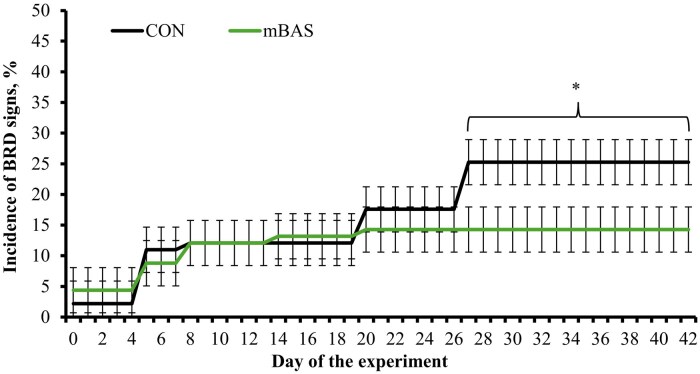
Cumulative incidence of bovine respiratory disease (**BRD**) signs during a 42-d feedlot period in bulls administered the maternal bovine appeasing substance (**mBAS**; *n* = 13; FerAppease; FERA Diagnostics and Biologicals; College Station, TX) or not (**CON**; *n* = 13) at the time of band-castration (d 0). the mBAS was applied topically to the nuchal skin area (5 mL) and above the muzzle (5 mL) of each bull, and reapplied on d 14 of the experiment. Cattle were observed daily for BRD signs including depression (lethargy, drooping ears), abnormal posture (gauntness, orthopnea), and altered respiratory character (nasal discharge, dyspnea). a treatment × day interaction was detected (*P* < 0.01). Within days, * *P* = 0.03.

A treatment × day interaction was detected (*P* < 0.01) for cumulative incidence of BRD signs, where mBAS cattle had reduced (*P* ≤ 0.05) incidence of BRD signs compared with CON beginning on d 27 of the experiment (Fig. 3). Overall, the proportion of cattle treated for BRD signs was less (*P* = 0.05) in mBAS compared with CON (Table 3). The proportion of cattle diagnosed with fever at first treatment also tended to be less in mBAS compared with CON (*P* = 0.07). No treatment effects were detected (*P* ≥ 0.33) within numbers of antimicrobial therapies required to regain health, and mortality rate did not differ (*P* = 0.32) between treatments. Castration is a stressful procedure that can impair immune function and increase susceptibility to BRD in feedlot bulls (Duff and Galyean 2007; Massey et al. 2011), which is consistent with the substantial incidence of BRD signs observed herein. Supporting our hypothesis and treatment effects on hair cortisol concentration, cattle administered mBAS had a 43% decrease in BRD signs and 45% decrease in fever at first treatment compared with CON. Pickett et al. (2024) reported similar BRD incidence in high-risk steers administered or not mBAS at arrival and 14 d later, but did observe that mBAS steers required fewer antimicrobial therapies and experienced reduced mortality during a 60-d feedlot receiving period. Kimbrough et al. (2025) administered mBAS to high-risk beef heifers and reported a 13.6% reduction in BRD incidence, but not in mortality during a 63-d feedlot receiving period. In Cooke et al. (2025), yearling *Bos indicus* bulls administered mBAS at feedlot arrival had a 53% decrease in morbidity associated with BRD, and a 64% decrease in mortality compared with non-treated bulls during a ∼100-d finishing period. Therefore, results from this experiment and previous studies demonstrate that the health-related responses to mBAS in high-stress cattle can be expressed differently depending on the production setting and BRD risk, but consistently result in improved outcomes such as reduced BRD incidence, enhanced antimicrobial therapy efficacy, or decreased mortality.

**Table 3:** Health parameters during a 42-d feedlot growing period of beef cattle administered the maternal bovine appeasing substance (**mBAS**; *n* = 13) or not (**CON**; *n* = 13).^a^^,^^b^

Item	CON	mBAS	SEM	*P-*value
***Cattle treated for signs of respiratory disease, %***	25.3	14.3	4.1	0.05
*** One treatment required to regain health***	68.5	58.6	14.4	0.65
*** Two treatments required to regain health***	22.0	38.3	11.2	0.33
*** Three treatments required to regain health***	4.20	7.99	5.80	0.65
***Cattle diagnosed with fever at first treatment, %***	22.0	12.1	3.95	0.07
***Mortality, %***	1.10	0.00	0.77	0.32

a Non-castrated bulls were purchased from 3 different sources with no previous health and management history, and transported to the research center on d –40. Bulls were assigned to initial processing and revaccination on d –36 and –12, respectively. Prior to initial castration on d 0, bulls individually received 10-mL of the mBAS (FerAppease; FERA Diagnostics and Biologicals; College Station, TX) or no treatment (CON). Treatments were applied topically to the nuchal skin area (5 mL) and above the muzzle (5 mL). Bulls were castrated using band-castration (Callicrate Pro-Bander^TM^; No-Bull Enterprises, LLC St. Francis, KS). The mBAS was reapplied to respective steers on d 14 of the experiment.

b Cattle were observed daily for signs of bovine respiratory disease (**BRD**) including depression (lethargy, drooping ears), abnormal posture (gauntness, orthopnea), and altered respiratory character (nasal discharge, dyspnea). Cattle diagnosed with these BRD signs received meloxicam (0.5 mg/kg of BW; Metacam, Boehringer Ingelheim, Ingelheim, Germany) and were assessed for rectal temperature. Cattle classified as febrile (rectal temperature ≥ 39.5°C) also received tulathromycin (Draxxin, Zoetis, Buenos Aires, Argentina) as their first antimicrobial therapy. Second and third antimicrobial therapies were florfenicol (Nuflor, Merck Animal Health, Madison, NJ) and enrofloxacin (Baytril, Bayer Animal Health, Shawnee Mission, KS).

### Behavioral responses

**Figure 4:**
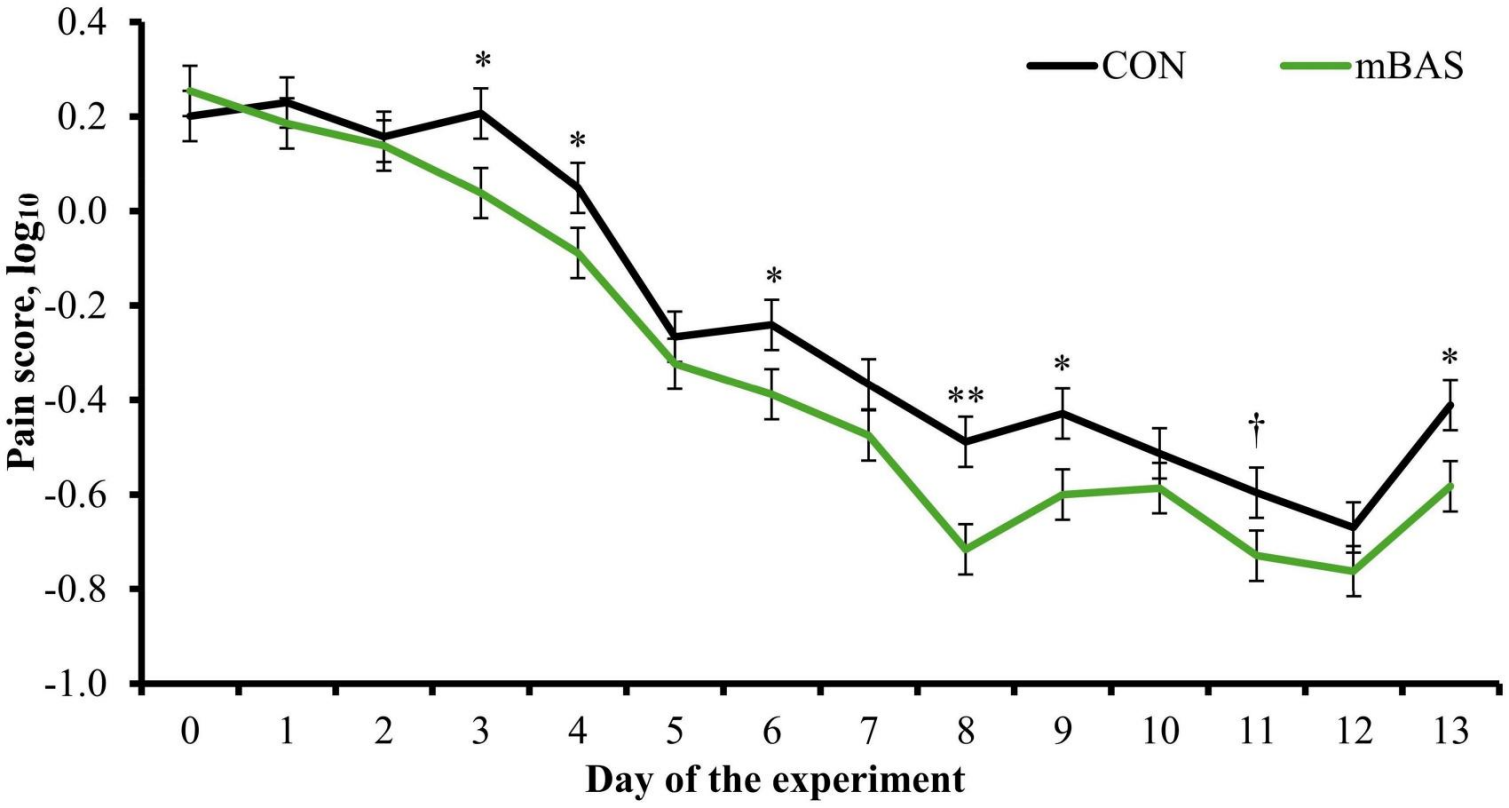
Pain score (log_10_ basis) during the 13-d following castration in feedlot bulls administered the maternal bovine appeasing substance (**mBAS**; *n* = 13; FerAppease; FERA Diagnostics and Biologicals; College Station, TX) or not (**CON**; *n* = 13) at the time of band-castration (d 0). the mBAS was applied topically to the nuchal skin area (5 mL) and above the muzzle (5 mL) of each bull, and reapplied on d 14 of the experiment. Subjective pain-related behaviors were evaluated daily from d 0 to 13 using 3-point scoring system (0 = no pain, 1 = moderate pain, 2 = severe pain) based on Gleerup et al. (2015). a treatment × day interaction was detected *(P* ≤ 0.01). Within days, ^†^*P* ≤ 0.10, *P ≤ 0.05 and ^**^P < 0.01.

A treatment × day interaction effect was detected for pain score (*P* ≤ 0.01), as mBAS cattle had reduced (*P* ≤ 0.05) pain score on d 3, 4, 6, 8, 9, and 13 compared with CON (Fig. 4). Moreover, mean pain score was also reduced (*P* ≤ 0.01) in mBAS cattle compared with CON (−0.224 vs. −0.331 log10, SEM = 0.019). Pain-related behaviors are validated indicators of post-castration discomfort in cattle (Gleerup et al. 2015; Tschoner and Feist 2022), indicating that mBAS administration lessened the behavioral expression of pain during the post-castration period rather than immediately following the procedure. However, this response should not be interpreted as a direct analgesic effect, but rather as mitigation of the stress response associated with the painful stimulus. In Pickett et al. (2024), mBAS administration reduced plasma cortisol but did not alter serum substance P concentrations following band-castration, suggesting attenuation of the stress response to the painful stimulus rather than inhibition of nociceptive signaling (Coetzee et al. 2008; Renthal 2020). The reduced pain scores observed herein, together with decreased hair cortisol concentrations, are consistent with attenuation of the adrenocortical response to castration (Stafford and Mellor 2011; Tschoner and Feist 2022) and support mBAS administration as a strategy to alleviate the behavioral and physiological consequences of this procedure in cattle.

**Figure 5:**
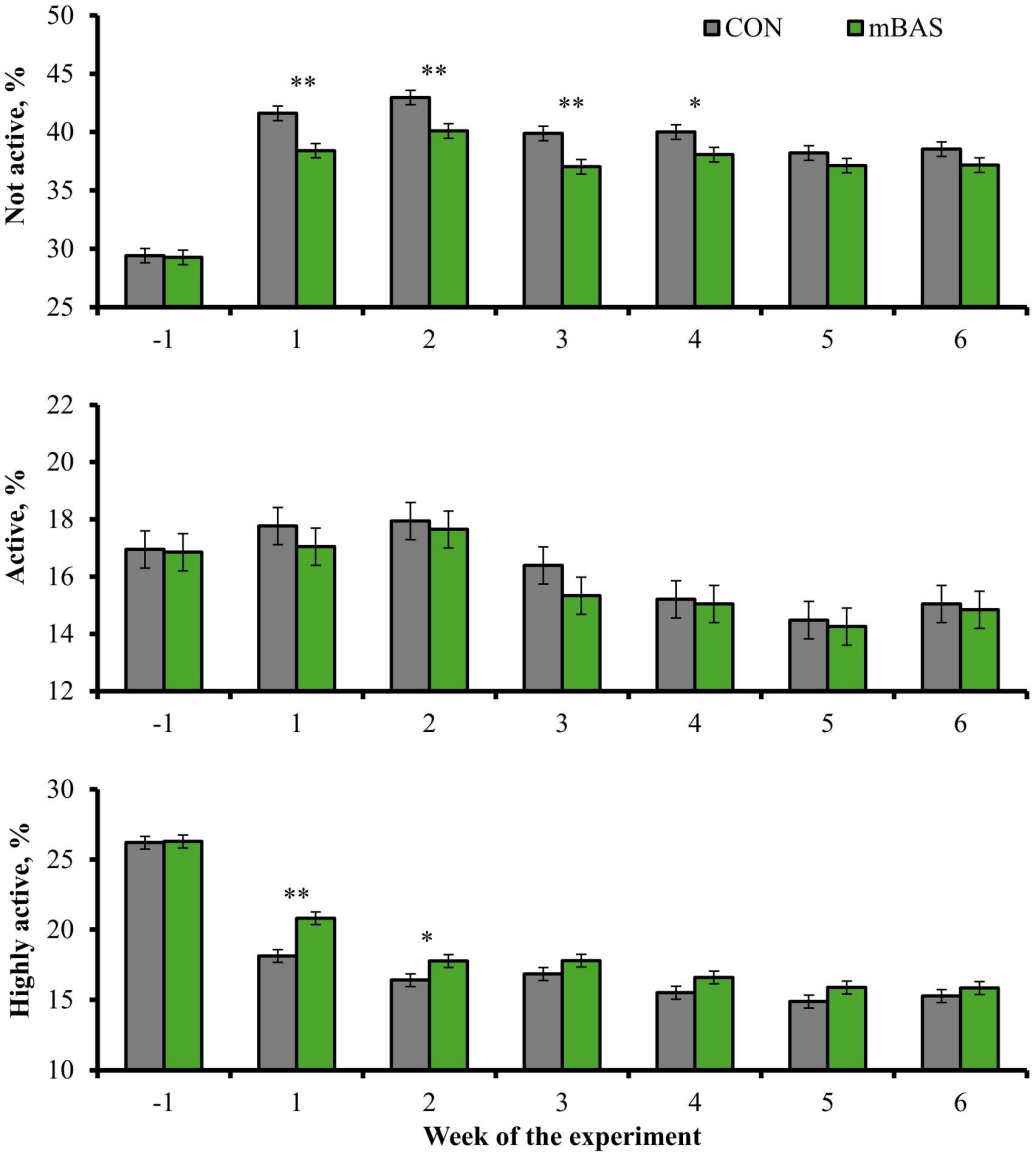
Physical activity (as % of time spent) during a 42-d feedlot period in bulls administered the maternal bovine appeasing substance (**mBAS**; *n* = 13; FerAppease; FERA Diagnostics and Biologicals; College Station, TX) or not (**CON**; *n* = 13) at the time of band-castration (d 0). the mBAS was applied topically to the nuchal skin area (5 mL) and above the muzzle (5 mL) of each bull, and reapplied on d 14 of the experiment. Activity parameters were evaluated as in Harvey et al. (2024) according to the CowManager system (Agis Automatisering BV, Harmelen, The Netherlands). Results are reported by week according to % of the time performing each activity. Treatment × day interactions were detected *(P* ≤ 0.04) for not active and highly active behaviors. No treatment effect nor treatment × day interaction were detected (*P* ≥ 0.15) for active behavior. Within days, ^†^*P* ≤ 0.10, *P ≤ 0.05 and ^**^P < 0.01.

**Figure 6:**
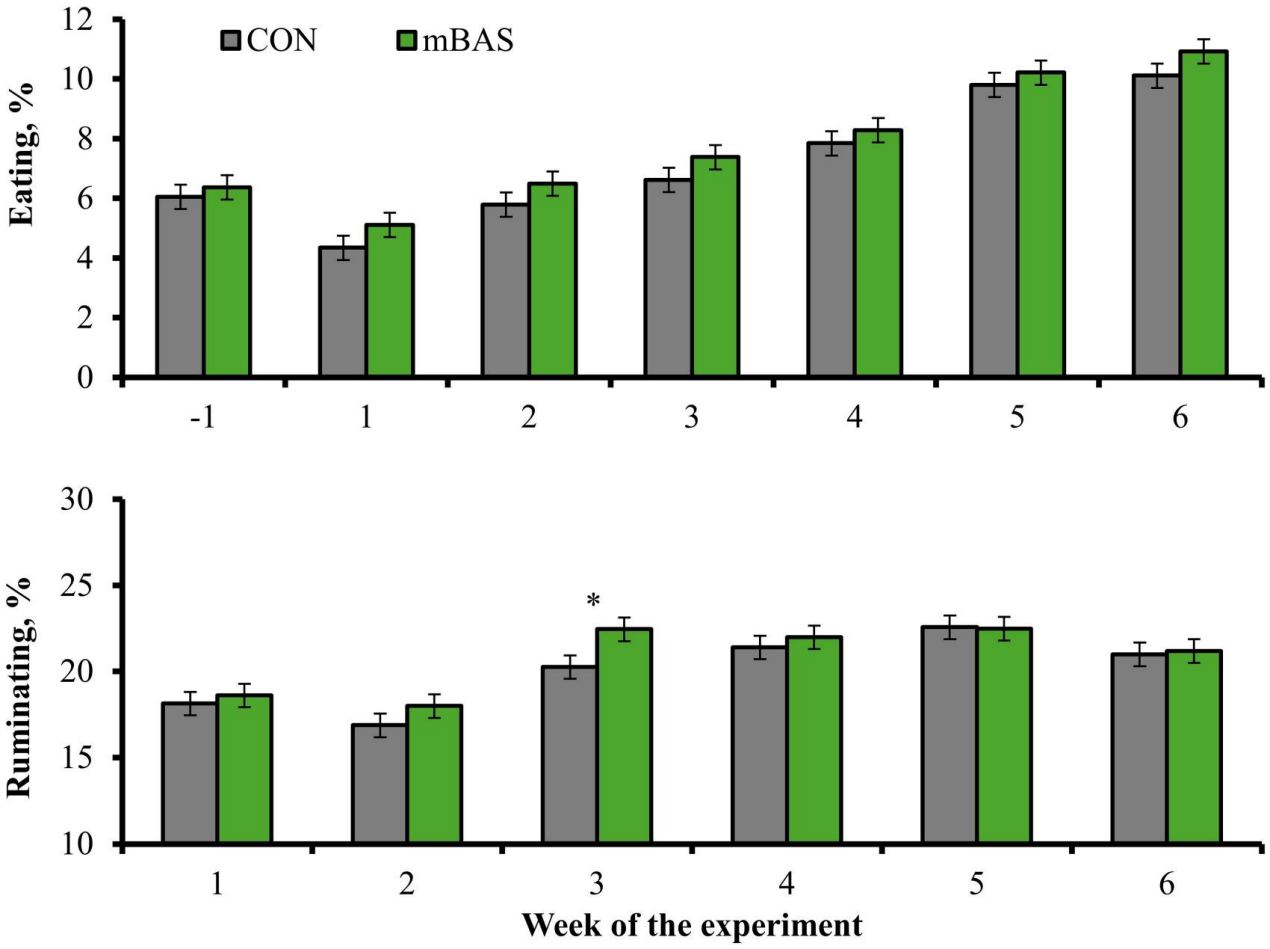
Physical activity (as % of time spent) during a 42-d feedlot period in bulls administered the maternal bovine appeasing substance (**mBAS**; *n* = 13; FerAppease; FERA Diagnostics and Biologicals; College Station, TX) or not (**CON**; *n* = 13) at the time of band-castration (d 0). the mBAS was applied topically to the nuchal skin area (5 mL) and above the muzzle (5 mL) of each bull, and reapplied on d 14 of the experiment. Activity parameters were evaluated as in Harvey et al. (2024) according to the CowManager system (Agis Automatisering BV, Harmelen, The Netherlands). Results are reported by week according to % of the time performing each activity. A treatment × day interaction was detected *(P* ≤ 0.01) for ruminating behavior. No treatment effect nor treatment × day interaction were detected (*P* ≥ 0.29) for eating behavior. Within days, *P ≤ 0.05.

Treatment × day interactions were detected *(P* ≤ 0.04) for time spent not active and highly active (Fig. 5), as well as time spent ruminating (Fig. 6). Cattle administered mBAS spent less time (*P* ≤ 0.03) being not active on wks 1 to 4, spent more time (*P* ≤ 0.03) being highly active during wks 1 and 2, and spent more time (*P* = 0.03) ruminating on wk 3 compared with CON cattle. No treatment effect or treatment × day interaction was detected (*P* ≥ 0.15) for time spent active (Fig. 5) and eating (Fig. 6). The similar time spent eating between treatments supports the lack of treatment differences for feed intake. The reduced inactivity observed in mBAS cattle during the early post-castration period is consistent with improved comfort and reduced stress, as increased inactivity is a common behavioral response to pain and disease (Weary et al. 2006; Tschoner and Feist 2022). Rumination is also associated with improved comfort, health status, and feed intake in cattle (González et al. 2008; Schirmann et al. 2009; Beauchemin 2018), although mBAS cattle spent more time ruminating during wk 3 only compared with CON. Hence, these behavioral responses are consistent with the reduced pain scores and decreased hair cortisol concentrations observed herein, and further support the benefits of mBAS administration in improving welfare in cattle following castration.

### Pen-based productivity responses

No treatment differences were detected (*P* = 0.21) for initial liveweight per pen (Table 4). Liveweight gain during the experiment tended (*P* = 0.09) to be greater for mBAS pens (Table 4). Hence, final liveweight on d 42 was greater (*P* = 0.05) in mBAS compared with CON pens (Table 4). Total feed consumed and G : F per pen during the experiment did not differ (*P* ≥ 0.15) between treatments (Table 4). Pens that received mBAS had greater (*P* = 0.05) final value and tended (*P* = 0.08) to have greater profit, whereas no treatment differences were detected (*P* ≥ 0.21) for initial value, feed costs, and medication costs per pen (Table 4).

**Table 4:** Productive and economical responses during a 42-d feedlot growing period of pens containing beef cattle administered the maternal bovine appeasing substance (**mBAS**; *n* = 13) or not (**CON**; *n* = 13).^a^

Item	CON	mBAS	SEM	*P-*value
***Productive responses***				
*** Initial liveweight,^b^ kg/pen***	2,146	2,161	8	0.21
*** Final liveweight,^b^ kg/pen***	2,257	2,308	17	0.05
*** Liveweight gain, kg/pen***	111.6	146.8	15	0.09
*** Total feed intake,^c^ kg/pen***	2,302	2,324	38	0.70
*** Gain to feed,^d^ kg/kg per pen***	0.049	0.064	0.007	0.15
***Economical assessment^e^***				
*** Initial value, $/pen***	19,160	19,296	73	0.21
*** Final value, $/pen***	19,094	19,521	143	0.05
*** Feed cost, $/pen***	691	697	11	0.70
*** Medication cost, $/pen***	53.4	38.2	8.5	0.23
*** Profit, $/pen***	–810	–511	118	0.08

a Non-castrated bulls were purchased from 3 different sources with no previous health and management history, and transported to the research center on d –40. Bulls were assigned to initial processing and revaccination on d –36 and –12, respectively. Prior to initial castration on d 0, bulls individually received 10-mL of the mBAS (FerAppease; FERA Diagnostics and Biologicals; College Station, TX) or no treatment (CON). Treatments were applied topically to the nuchal skin area (5 mL) and above the muzzle (5 mL). Bulls were castrated using band-castration (Callicrate Pro-Bander^TM^; No-Bull Enterprises, LLC St. Francis, KS). The mBAS was reapplied to respective steers on d 14 of the experiment.

b Sum of the body weight within each pen recorded on d 0 (initial) and d 42 (final); cattle removed during the experiment (mortality) were assigned a final BW of zero.

c Evaluated by recording daily offer and measuring refusals daily from each pen; thus, feed intake includes consumption by all animals prior to removal.

d Calculated using liveweight gain and total feed intake (kg of dry matter) of each pen during the 42-d experimental period.

e Initial and final value calculated using pen liveweight added a 4% shrink, and $9.30/kg for initial and $8.81/kg for final liveweight value. Feed cost was estimated at $300/ton, and medication cost as $22 for first therapy when fever was diagnosed, $12 for first therapy when fever was not diagnosed, $35 for the second therapy, and $9 for the third therapy. The labor required for mBAS application was considered negligible, as treatments were administered during routine handling procedures and did not require additional processing time. The profit of each pen was estimated as final value – (initial value + feed costs + medication costs).

Similar to Pickett et al. (2024), the increase in pen-based liveweight gain in mBAS pens was primarily driven by numerical reductions in mortality, which accounted for most of the difference in total liveweight gain per pen. Based on the magnitude of treatment differences, a smaller proportion of this response was associated with numerical improvements in ADG. The reduced incidence of BRD observed in mBAS cattle likely contributed indirectly to these differences by minimizing performance losses associated with morbidity (Pickett et al. 2023). Therefore, mortality was the primary determinant of pen-based productivity, whereas morbidity-related responses contributed secondarily through maintenance of ADG. Current U.S. cattle, feed, and medication prices were used to facilitate interpretation of the pen-based productivity responses; however, these values vary according to time and location and should be interpreted with caution. Although medication costs did not differ statistically between treatments, the numerical reduction in BRD incidence and fever at first treatment in mBAS cattle also contributed to the tendency for increased profit. Hence, mBAS administration resulted in a return-on-investment (**ROI**) of 714%, calculated as the difference in profit between CON and mBAS pens ($–810 vs. $–510; $300 decrease in loss) divided by the cost of mBAS application per pen ($42; $3 per 10-mL dose, 7 animals per pen receiving 2 doses). This pen-based assessment provides evidence that the health and welfare benefits associated with mBAS administration during castration can be translated into improved productivity under commercial feedlot conditions.

### Overall conclusions

Administering mBAS to feedlot-adapted bulls at the time of castration attenuated chronic stress, reduced the behavioral expression of pain, and decreased the incidence of BRD during the 42-d post-procedural period. Castration of heavier cattle after feedlot entry is a common practice in South American systems, and represents a relevant welfare and health challenge. The improvement in pen-based productivity from mBAS administration resulted from greater liveweight/pen, and was associated with reductions in morbidity and numerical decreases in mortality. The benefits of mBAS were primarily expressed through improved health and welfare rather than enhanced individual performance as in Pickett et al. (2024). This experiment provides additional evidence that the biological responses to mBAS in high-stress cattle are consistently manifested as improved stress mitigation, health, and overall productivity, whereas the specific performance variables affected may vary according to the production scenario and BRD risk.

## Disclosures

Martin Zinicola is a partner of Cowix (San Francisco, Córdoba, Argentina), which distributes FerAppease in Argentina. The other authors of this manuscript have no conflict of interest to report. Pheromone-based products, including those marketed as bovine appeasing substances, are not currently subject to standardized regulatory definitions, and substantial variation may exist among commercial formulations with respect to composition, concentration, and vehicle. Such differences may influence biological responses. Therefore, the outcomes reported herein are specific to the FerAppease formulation evaluated and should not be extrapolated to other products marketed under similar terminology without independent validation.

## List of abbreviations

ADG: average daily gain
BRD: bovine respiratory disease
BW: body weight
G : F: gain to feed
mBAS: maternal bovine appeasing substance
ROI: return-on-investment
TMR: total mixed ration
